# Explaining Adherence Success in Sub-Saharan Africa: An Ethnographic Study

**DOI:** 10.1371/journal.pmed.1000011

**Published:** 2009-01-27

**Authors:** Norma C Ware, John Idoko, Sylvia Kaaya, Irene Andia Biraro, Monique A Wyatt, Oche Agbaji, Guerino Chalamilla, David R Bangsberg

**Affiliations:** 1 Harvard Medical School, Boston, Massachusetts, United States of America; 2 Jos University, Jos, Nigeria; 3 Muhimbili University of Health and Allied Sciences, Dar es Salaam, Tanzania; 4 Mbarara University of Science and Technology, Mbarara, Uganda; 5 Muhimbili University/Dar es Salaam City Council/Harvard School of Public Health HIV/AIDS Care and Treatment Program, Dar es Salaam, Tanzania; 6 Harvard School of Public Health, Boston, Massachusetts, United States of America; 7 Massachusetts General Hospital, Boston, Massachusetts, United States of America; 8 Harvard Initiative for Global Health, Cambridge, Massachusetts, United States of America; Johns Hopkins University, United States of America

## Abstract

**Background:**

Individuals living with HIV/AIDS in sub-Saharan Africa generally take more than 90% of prescribed doses of antiretroviral therapy (ART). This number exceeds the levels of adherence observed in North America and dispels early scale-up concerns that adherence would be inadequate in settings of extreme poverty. This paper offers an explanation and theoretical model of ART adherence success based on the results of an ethnographic study in three sub-Saharan African countries.

**Methods and Findings:**

Determinants of ART adherence for HIV-infected persons in sub-Saharan Africa were examined with ethnographic research methods. 414 in-person interviews were carried out with 252 persons taking ART, their treatment partners, and health care professionals at HIV treatment sites in Jos, Nigeria; Dar es Salaam, Tanzania; and Mbarara, Uganda. 136 field observations of clinic activities were also conducted. Data were examined using category construction and interpretive approaches to analysis. Findings indicate that individuals taking ART routinely overcome economic obstacles to ART adherence through a number of deliberate strategies aimed at prioritizing adherence: borrowing and “begging” transport funds, making “impossible choices” to allocate resources in favor of treatment, and “doing without.” Prioritization of adherence is accomplished through resources and help made available by treatment partners, other family members and friends, and health care providers. Helpers expect adherence and make their expectations known, creating a responsibility on the part of patients to adhere. Patients adhere to promote good will on the part of helpers, thereby ensuring help will be available when future needs arise.

**Conclusion:**

Adherence success in sub-Saharan Africa can be explained as a means of fulfilling social responsibilities and thus preserving social capital in essential relationships.

## Abstract 2

Using ethnographic data from Nigeria, Tanzania, and Uganda, Norma Ware and colleagues examine why levels of adherence to HIV/AIDS drugs are so much higher in sub-Saharan Africa than in North America.

## Editors' Summary

**Background.:**

The acquired immunodeficiency syndrome (AIDS) epidemic has killed more than 25 million people since 1981, and about 30 million people (22 million in sub-Saharan Africa alone) are currently infected with the human immunodeficiency virus (HIV), which causes AIDS. HIV destroys immune system cells, leaving infected individuals susceptible to other infections. Early in the AIDS epidemic, most HIV-infected individuals died within ten years but in 1996, combination antiretroviral therapy (ART)—a mixture of powerful drugs—was developed. For HIV-infected people living in affluent, developed countries, HIV/AIDS became a chronic disease, but for the millions of infected people living in low- and middle-income countries, HIV/AIDS remained a death sentence—ART was simply too expensive. In 2003, this situation was declared a global health emergency. Today, through the concerted efforts of governments, international organizations, and funding bodies, nearly one-third of the people in developing and transitional countries who are in immediate need of life-saving ART receive free, reliable supplies of the drugs they need.

**Why Was This Study Done?:**

For ART to work, it must be taken regularly. If drug doses are missed, the virus can rebound and resistance to ART is more likely to develop. In poor countries, even though free antiretroviral drugs are increasingly available, many obstacles to good adherence to ART remain. These include economic obstacles (for example, the cost of traveling to clinics and the loss of earning associated with clinic attendance), and social, cultural, and behavioral barriers. Some patients fear disclosure, for example. Others receive conflicting messages about the benefits of ART. However, despite worries that the scale-up of ART provision in developing countries would be dogged by inadequate adherence, people living with HIV/AIDS in sub-Saharan Africa generally take more than 90% of their prescribed doses of ART, a better level of adherence than in North America. In this study, the researchers investigate why ART adherence is so high in sub-Saharan Africa by analyzing qualitative data from an ethnographic study done in Nigeria, Tanzania, and Uganda. Qualitative data are often used to address “how” and “why” research questions: ethnography is a comprehensive qualitative approach to describing and explaining human behavior and culture.

**What Did the Researchers Do and Find?:**

For their study, the researchers interviewed 158 patients, 45 treatment partners (lay-people who help HIV-positive people keep to their treatment), and 49 health care workers. Patients were asked about their experiences of ART and about the help they received from their treatment partners; partners were asked about the type of help they gave and about their feelings about this help; health care workers were asked to describe a typical clinic visit and to indicate how adherence was discussed. From these interviews and observations of clinic sessions, the researchers identified several strategies used by patients and their treatment partners to overcome economic obstacles to ART adherence. These included borrowing and “begging” funds to pay for travel to clinics and making “impossible choices” to prioritize adherence, and “doing without.” The researchers' analysis also indicates that the prioritization of adherence to ART reflects the importance of relationships as a resource for managing economic hardship. So, for example, they found that treatment partners and health care workers expected patients to adhere to ART (which, by improving patients' health, improves their ability to support themselves and their families) and made their expectations known, thereby creating a responsibility among patients to adhere. Patients, in turn, adhered to their treatment to promote good will from their helpers and thus ensure their continuing help.

**What Do These Findings Mean?:**

The findings offer a possible explanation of adherence success in sub-Saharan Africa. The high level of adherence to ART can be explained as a means of fulfilling social responsibilities. Adherence, the researchers suggest, not only improves personal health (the main driver for ART adherence in resource-rich environments) but also preserves “social capital” in essential relationships. In other words, in sub-Saharan Africa, adherence to treatment may protect the relationships that individuals living in extreme poverty rely on to help them survive.

**Additional Information.:**

Please access these Web sites via the online version of this summary at http://dx.doi.org/10.1371/journal.pmed.1000011.

- This study is further discussed in a *PLoS Medicine* Perspective by Agnes Binagwaho and Niloo Ratnayake
- Information is available from the US National Institute of Allergy and Infectious Diseases on HIV infection and AIDS
- HIV InSite has comprehensive information on all aspects of HIV/AIDS, including an article about to antiretroviral therapy
- Information is available from Avert, an international AIDS charity, on HIV and AIDS in Africa (including detailed information on HIV/AIDS in Nigeria and Uganda) and on providing AIDS drug treatment for millions
- The World Health Organization provides information about universal access to HIV treatment (in several languages)
- The US Centers for Disease Control and Prevention also provides information on global efforts to deal with the HIV/AIDS pandemic

## Introduction

People living with HIV/AIDS in sub-Saharan Africa generally take more than 90% of prescribed doses of antiretroviral therapy (ART) [1–12]. This number exceeds the levels of adherence observed in North America [13] and dispels early scale-up concerns that adherence would be inadequate in settings of extreme poverty [14–16].

These near-perfect levels of adherence are being achieved despite formidable obstacles in the poorest regions of the world. Prohibitive costs of self-pay medications and drug stock-outs create insurmountable access barriers and lead to missed doses [3,7,10,12,17–20]. Expanding access to free ART and better distribution systems are addressing these obstacles. However, out-of-pocket expenses, including user fees, laboratory tests, and transportation over often long distances to and from treatment sites remain important barriers to sustained adherence and medical care [5,21–24]. Time spent traveling to and attending clinical appointments places additional economic strain on patients and their families by competing with income generating activities. Failure to negotiate economic obstacles can lead to ART interruptions, viral rebound, and resistance to limited available regimens [2,25].

Social and cultural obstacles also threaten adherence success. Stigma and the fear of disclosure cause patients to skip doses if privacy is unavailable at a scheduled dosing time [5,21]. Conflicting messages from health care practitioners and religious authorities may also interfere with adherence [26,27]. Behavioral obstacles similar to those observed elsewhere, including forgetting doses, fear of side effects, traveling without medications, and stopping drugs when symptoms disappear have also been reported for sub-Saharan Africa [3,4,10,18,25,26].

Adherence facilitators in resource-scarce settings have received less attention than adherence barriers. Currently, home-based adherence “help” is emerging as an important resource to support adherence. One form of home-based help centers on community health workers. Community health workers are laypersons trained and paid through treatment programs to provide adherence support and other services at home. They deliver medications, observe dosing, refer individuals for HIV-testing, offer encouragement, provide nutritional and housing support, help with transportation, and in general represent important links between clinics and communities [22,28–32].

A second form of home-based help is treatment supporters [33]. Treatment supporters (also termed treatment “partners” and treatment “assistants”) are laypersons nominated by patients to help with treatment adherence. They remind patients of dosing times, and often (though not always) witness dosing. They are not health care workers, but individuals with close personal ties to patients. As family members or friends, treatment supporters often live in the same or a nearby household. Treatment supporters are not specifically trained or paid, but nonetheless make a formal commitment to their role. Early studies suggest that both treatment support and community health worker approaches to home-based help may improve treatment outcomes [4,22,28,34].

More structured support in the form of clinic-based modified directly observed therapy (MDOT) has shown promising results in Kenya [35]. The factors accounting for the observed benefit of MDOT are currently unknown. Results of similar initiatives in the United States are more mixed [36,37].

To better understand exceptional adherence in sub-Saharan Africa, we analyzed qualitative data from an ethnographic study carried out in three countries: Nigeria, Tanzania, and Uganda.

## Methods

### Study Design

This patient-centered ethnographic study took place in public HIV-treatment settings in Nigeria, Tanzania, and Uganda (see Table 1). The HIV/AIDS Clinic at Jos University Teaching Hospital (JUTH) is located in a medium-sized city in Nigeria's central plateau and currently follows more than 8,400 patients prescribed ART. The ART Clinic of Amana District Hospital is located in Dar es Salaam, Tanzania, and currently follows approximately 5,300 patients on ART. The Immune Suppression Syndrome (ISS) Clinic at Mbarara University of Science and Technology is located in rural southwest Uganda and currently follows approximately 7,000 patients prescribed ART. A wide range of geographic and social variation is represented across the three sites.

**Table 1:**
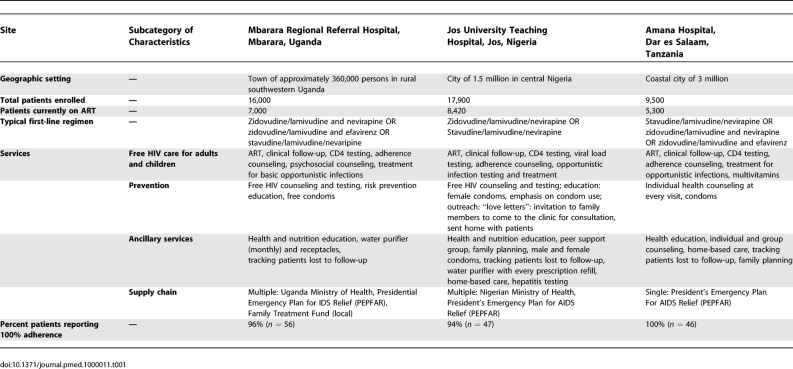
Study Site Characteristics

### Sampling

We used a purposeful sampling design. The goal of purposeful sampling is to systematically represent a variety of perspectives on the topic under study [38]. Three different perspectives chosen for relevance to the topic of adherence were represented in this research: (1) patients; (2) treatment partners (termed “assistants” at the Tanzanian site); and (3) health care providers. In Nigeria and Tanzania, random samples of potential patient participants were drawn from the larger populations of adults meeting inclusion criteria. Inclusion criteria were: (1) age 18 y or older; (2) prescribed antiretroviral therapy for HIV/AIDS for no fewer than 6 and no more than 12 mo at the time of sampling; and (3) residence within 20 km of Jos University Teaching Hospital or Amana District Hospital. In Uganda, patient participants were drawn from the larger Uganda Antiretroviral Treatment Outcomes (UARTO) study, a prospective study of antiretroviral-naïve patients initiating ART led by one of the authors (DRB).

Treatment partners who participated in the study were referred by patient participants. Patients were asked to name “someone who assisted them in their efforts to take antiretroviral medications.” Health care providers who participated were volunteers who responded to a letter of invitation introducing the study.

### Recruitment

In Nigeria and Tanzania, patient participants were recruited at the clinic during routine follow-up visits. At a free moment (e.g., while waiting to be seen or at the conclusion of the visit), staff approached eligible individuals to describe the study and extend an invitation to participate. In Uganda, permission to contact prospective patient participants for recruitment was first obtained by UARTO research staff. Once permission was obtained, a research assistant for this ethnographic study approached the participant for consent.

Treatment partners were visited or telephoned by staff after giving permission for contact. Health care providers who indicated interest in response to the letter of invitation were invited to participate.

The research was approved by the institutional review boards at Harvard Medical School (Boston, Massachusetts, United States); Jos University Teaching Hospital (Jos, Nigeria); Muhimbili University of Health and Allied Sciences (Dar es Salaam, Tanzania); Mbarara University of Science and Technology (Mbarara, Uganda); and the Uganda National Council for Science and Technology (Kampala, Uganda). Written informed consent was obtained from all participants.

### Data Collection

Data were collected through in-person qualitative interviews and observations of clinic activities. Information on adherence was collected through the 3-d self-report instrument from the AIDS Clinical Trials Group edited and adapted for cultural context [39]. All data were collected by African researchers. The researchers were Nigerians, Tanzanians, and Ugandans who had been trained in ethnographic data collection techniques by two of the authors (NCW and MAW).

### In-Person Interviews

Multiple interviews were conducted with patient and treatment partner participants to allow for elaboration of areas whose significance emerged as analysis proceeded. The interviews were semistructured, meaning that predesignated core topics, but not specific questions, were covered in each session. This approach ensured that specific areas were covered in each interview, but also allowed unanticipated themes to emerge. Core topics for patient interviews included: (1) specific experiences of taking ART (e.g., “stories” of the most recent dose taken, most recent dose missed); (2) clinic visits; (3) help received from treatment partners. Core topics for treatment partner interviews included: (1) types of help provided; (2) feelings about being a treatment partner; (3) perceptions of the impact of help. Core topics for health care provider interviews included: (1) description of typical clinic visit; (2) the ways adherence comes up in clinical visits; (3) perceptions of barriers to adherence for patients.

The goal of the interviews was to understand ART adherence from the interviewees' points of view. Patient participant and treatment partner interviews were conducted in homes or at other locations of the interviewee's choosing outside the clinic. Health provider interviews were conducted at the clinic. Privacy was protected by conducting the interviews in locations where the conversation could not be overheard. Interviewees had the option of conducting the interview in the local language (Hausa, Kiswhahili, Runyankore) or in English. Interviews were audio-recorded with permission and averaged about an hour in length. Patients received compensation in the form of reimbursement for transportation (where applicable) or a small stipend.

### Field Observations

Observations are a hallmark of ethnographic data collection. They involve the presence of a researcher in a naturalistic setting and the witnessing of events and activities of interest to a given research inquiry [40]. Observations have the advantage of providing a direct view of phenomena under study to complement one that is mediated through verbal interview reports. The following activities were observed by research staff at each of the clinical sites: (1) routine follow-up visits of patients taking ART; (2) counseling sessions; (3) health education sessions; and (4) the dispensing of antiretroviral medications. Observation sessions lasted 30 min to 1 h.

### Data Preparation

Shortly after the completion of each interview, the interviewer produced a detailed write-up in English on the interview content, using the audio-recording and notes taken during the interview to ensure accuracy and completeness. The interview write-ups took the form of “stream-lined” transcripts. A “stream-lined” transcript is produced in English (without first transcribing in the local language) and consists only of interview questions asked and their responses. This approach to data preparation has several advantages. First and foremost, it captures the desired level of detail often lost in a summary and preserves the exact words of the interviewee. It can be completed in a reasonable amount of time by the interviewer, eliminating the expense of separate transcription. Accuracy is also improved using this approach. The write-ups contain a section in which the interviewer adds relevant contextual details and impressions not captured in the transcript. Descriptions of activities observed were written up following each session as field notes.

### Data Analysis

Analysis aimed at explaining adherence to ART using an inductive approach to category construction and interpretation of data [41,42]. Descriptive categories were constructed to characterize approaches to overcoming economic obstacles to care. To construct the categories, content related to patients' adherence experiences was retrieved from coded data. Relevant sections of text were identified, copied, and grouped according to the type of adherence obstacle represented. These sections of text were then reread to characterize the ways in which patients negotiated these obstacles to avoid missing doses and clinic visits. The data were re-organized in terms of these characterizations to produce an initial category “set.” Each category forming the set was named, defined, and illustrated through interview excerpts. The set was refined—revised, specified, and elaborated—through successive returns to the data in which additional sections of relevant text were extracted. Interpretive analysis used a theoretic social science construct to develop an argument based on the categories. The data suggested “social capital,” i.e., resources accruing from social networks, as the best explanatory construct to interpret adherence strategies and explain their success.

## Results

### Study Participants

158 patients, 45 treatment partners, and 49 health care providers participated in this study. 414 interviews and 136 observation sessions were conducted across the three sites.

### Characteristics of Study Participants

#### Patients.

Approximately two-thirds (65%) of patients who participated in this study were women. The mean age of the patient group was 38 y. Less than half (44%) were married or living with a partner. Approximately three-quarters (71%) of the patients were Christian; the remainder were Muslim. Primary school was the highest level of education completed for slightly more than half (57%) of the patient sample. 65% of patients met criteria for Stage III or Stage IV disease according to World Health Organization (WHO) criteria at initiation of HIV care.

#### Treatment partners.

More than half (56%) of treatment partners were female. The mean age of the group was 39 y. Three-quarters (73%) were married or living with a partner. 60% were Christian; the remainder were Muslim. Primary school was the highest level of education completed for about a third (35%) of the group.

#### Health care providers.

Approximately two-thirds (64%) of health care provider participants were women, and the mean age of the group was 38 y. About a quarter (27%) of the sample were physicians; a third (35%) were nurses. Nineteen percent were counselors and 16% were pharmacists or medication dispensers. Two full-time “tracking” officers, responsible for locating patients lost to follow-up, were included in the sample (2%), as was another expert patient fulfilling an administrative function (1%).

### Ethnographic Data

On the basis of category construction and interpretation of the data, we organized the findings from ethnographic data to demonstrate: (1) prioritization of adherence to overcome economic obstacles, and (2) responsibility in social relationships as an explanation of prioritization.

#### Prioritizing adherence to overcome economic obstacles.

Participants reported that most adherence obstacles were related to resource scarcity. Resource scarcity means that basic commodities required for daily living are not easily available. For example, obtaining money for transportation to clinic appointments (where prescriptions are refilled) is rarely a simple matter of dipping into a ready supply of cash. Concerted effort and specific strategies were required to raise needed funds to secure medications.

Patient participants spoke of raising transport funds through loans and handouts, what they termed borrowing and “begging.” Loans—from treatment partners and other family and friends—were a reliable source of cash but were expected to be repaid, often with interest. Some interviewees reported selling possessions or earning income through work to repay loans. Others borrowed repeatedly but saw no way to resolve the debts they had incurred. Chronic indebtedness was a source of considerable emotional distress for borrowers, as these interview excerpts indicate: “Most times I borrow money [to get to clinic] and worry about re-paying the debt.” (*Interviewer:* “How do you re-pay the debts?”) “I work and look for money digging and weeding in people's plantations.” And: “When you do not have money you think of how you will come to clinic for medicine. [Last time] I borrowed from a friend but now I have paid it back.” (*Interviewer*: “How did you feel about borrowing?”) “I felt raw and depressed because I got worried. Actually I lost kilograms. When I came to the clinic and got weighed I had lost 2 kgs.”

Treatment partners reported borrowing to help patients. In the following excerpt, a treatment partner describes how “having friends who have shops” provides access to credit that enables her to accommodate the food preferences of the patient she helps, who is not doing well: “The patient is seriously sick now. She is not ok. And when patients are seriously sick they lose their appetite. If you give her beans she will not eat. So we are borrowing rice from people with shops. They trust us and they lend, paying is a problem. I live with good neighbors who have shops. They help me, and at the end of the month I pay them. She likes rice very much. If you cook rice with anything, she eats.”

Requesting handouts of cash—termed “begging” by interviewees—was also reported. Handouts were not expected to be repaid. Interviewees “begged” handouts for transport funds from family, friends, and health care providers: “If I see the date [of clinic visit] is approaching, I will start begging for help. I tell people the date of my clinic is coming and that I don't have money, so they should assist me.” And: “Sometimes I beg or ask for money to attend the clinic. Sometimes I have money for going there and beg at the clinic [for money to return]. Last time my uncle gave me money to go there. To get back I had to beg for money from the doctor.”

Cash in hand, whether borrowed, “begged,” or earned, is immediately subject to competing claims. As a result, patient participants were continually confronted with “impossible choices”: HIV care or treatment for a sick child? HIV care or school fees? HIV care or food for the family? Resource scarcity sometimes required denying the needs of loved ones in order to secure money for transportation to clinic: “Seven days before going to get the medicine, I try to look around for money. If it means not eating or buying food for the children I do that, because they told us that we are not supposed to miss …”

Food, essential to survival and functional recovery while on ART, was often treated as expendable. Patient participants understood that taking medications with food and adhering to a healthy diet that includes a variety of food groups is important for their treatment and recovery on ART. Though reluctant to ignore doctors' orders, patient participants sacrificed their own need for food to meet the needs of others close to them: “If I don't have porridge [when I take the pills] I feel nauseated. [But at times it's hard to get porridge] because I buy it with money and when it's the school fee season there is no money.”

Strategies for obtaining help from others notwithstanding, there are times when patients simply “do without.” They do without food, but take their medications anyway, despite hunger-induced exacerbation of ART side effects. They do without transport, and walk to clinic, which can often take several hours: “It's hard to take meds without food. When I take meds without food I get a stomachache. But you can't miss meds because of food. I take meds with an expectation that I may get food later.” And: “[When there's no food] I just take [the drugs] on an empty stomach. And I will feel like vomiting – dizzy, weak and all. When I don't eat before taking drugs, honestly, I don't find it easy at all.” And: “Five months ago the car got spoilt and now I am walking to the clinic. At first I had money for a [motorcycle taxi] but now I do not even have that. I will continue walking whatever happens.”

Borrowing and “begging” transport funds, making impossible choices, and “doing without” are ways of overcoming the economic obstacles that continue to block access to treatment for HIV/AIDS despite the availability of free ART. These sacrifices reflect the extraordinary efforts and commitment of individuals who are assigning “first priority” to HIV care: “Taking meds is my life. I have to give meds first priority because they are helping me.”

#### Explaining prioritization: adherence to improve health and fulfill responsibility in social relationships.

As in resource-rich settings, the immediate reason patients assign “first priority” to adherence is to improve health. Patient participants described profound improvements after starting ART, as grave illness gave way to weight gain, renewed energy and strength, and the ability to care for themselves. With treatment, most patients returned to normal activities, including income-generation. Clinical improvement also brought renewed hope for a long life. The restoration of health and hope, together with the memory of previous suffering and the threat of jeopardizing what has been gained should adherence lapse, provide powerful motivation to take ART correctly.

However, the pursuit of health does not completely explain why patients assign first priority to adherence. For African patients, good health is important because it helps to preserve relationships with others.

The contribution of social relationships to mental and physical health is well-documented for North America [43–47]. In settings of economic scarcity, relationships are not only important for health but also essential to survival. Our data suggest the prioritization of adherence reflects the importance of relationships as a resource for managing economic hardship.

Chronic illness is always hard on close relationships. But when health care is a scarce resource, illness imposes an extra burden on social intimates who must then assume responsibility for care. Care provision means investing valuable time, energy, and financial resources to promote the patient's health. The investment is justified when there is an expectation of recovery, but becomes harder to defend if it appears the illness may be terminal. When an illness is considered terminal, caregivers may look for ways of severing their connection with the patient and ending the drain on scarce resources. One interviewee explained it this way: “When I fell sick, some of the people in my family thought I was going to die soon, so they advised my brother and sister (whom I was living with) to look for transport to send me back to the village. Some people, if they see you in terminal illness, they start to value their money. They think, ‘this illness is getting worse. It'll really cost me to transport a dead body, whereas to arrange to transport a living body is different….'”

Patient participants in this study received adherence assistance from several sources. In addition to money for transport, treatment partners, other family and friends, and fellow patients provided regular reminders of dosing times. They offered encouragement by emphasizing the benefits of treatment and continually reinforcing the importance of taking medications as prescribed. Treatment supporters also worked to destigmatize HIV/AIDS. They socialized in public with infected persons; likened HIV/AIDS to common, but less stigmatized conditions (e.g., malaria); and challenged myths by deliberately sharing food and eating utensils. “…when they [a set of friends of the patient] started understanding the problem [HIV/AIDS], everybody started withdrawing from eating in the same container with him. …I saw everyone was running away from him so I called him and lied to him. I did not want to create division and break him the more. So I told him, ‘look, I met one of your doctors and she told me that you have to be very careful because of infection. You don't eat anything anybody else eats, you don't use the same cup anybody else uses, because you could get another infection and it could be even worse.' And then I ate with him, so the others would see.”

Health care providers were also important sources of help. They supported adherence through education, reinforcement, even the organization of care. At one site, providers maintained a flexible schedule to accommodate patients, even though it made their days longer. They instituted a policy of accepting “latecomers”—individuals who arrived after the deadline for being seen on a given day had passed.

Not infrequently, providers stepped outside their professional roles to make resources available to those in need. They provided money and food at their own expense, and organized larger efforts to generate support. In the following quote, a provider describes her practice of helping indigent women patients toward financial independence by securing small loans and then indicating how they might use the funds to start small businesses: “I say, ‘go around in your area, your community. Look at what people like that is not available there. Go and get some of these things and begin to sell them.' That is how I do it.”

Providers expected adherence and did not hesitate to make their expectations known. In clinic, staff communicated expectations of adherence by stating repeatedly that antiretroviral therapy must be taken as prescribed. In extreme cases, they even threatened those who repeatedly missed clinic appointments with withdrawal of access, as we see from the following report: “I told [the patient] this morning, ‘You had better decide if you are going to take these drugs. Because the way you are doing, you are creating resistance. I am giving you my last warning. If you come next time with the same story, even if you beg from heaven, we will discontinue you.' That is what I told her. (*Interviewer:* And would you really?) If she comes back with the same story, I am serious. We will discontinue her. We will.”

Treatment partners also expected adherence. As one person put it: “We insist she take her meds. We help her because we want her to take her meds….If you don't take your meds, you will die.”

Friends and family members taking responsibility for someone's well-being were sensitive to the fact that adherence meant “doing well,” and “doing well” made their job easier. They insisted on adherence as a gesture aimed at reducing what one treatment partner termed the “work” of care. He explained this way: “If he [patient] continues well, the work of caring for him will be over. If he continues well, I can visit him at the time I want. But if he is sick, I have to help him so he will be okay and everyone else can continue with their business. That's why I insist, ‘my relative, don't ignore what they instruct you to do. If they tell you to take [ART] in the morning and evening—do it! Don't feel it is difficult work and don't feel tired.'”

Making a similar observation, someone else said: “[Before the patient improved] it was difficult. I had to be there all the time. I kept the time for meds in my mind instead of him. I was unable to go anywhere or do anything else before he takes his meds. Now I'm free to do anything at any time without having many thoughts.”

Social expectations of adherence create obligations on the part of patients, who must meet these expectations to preserve relationships with helpers. By taking ART as prescribed and “doing well,” patients reduce the burden of help and acknowledge the work of care. This in turn fosters continued good will on the part of helpers and reinforces the likelihood that assistance will be available when future needs arise. In settings of poverty, where “community safety nets” [48] replace public entitlements as resources for weathering economic crises, the consistent good will of potential helpers is required for survival.

## Discussion

Our data point to economic obstacles and the strength of social relationships as principal mediators of sustained adherence in sub-Saharan Africa. These obstacles are being routinely overcome through strategies aimed at prioritizing adherence. Prioritization is accomplished with help from others. The relationships that provide this assistance are a critical resource not only for supporting adherence, but for managing economic hardship more generally. In social science, the use of relationships to obtain benefits and achieve desired ends has been termed “social capital.”

In North America, adherence to ART for HIV/AIDS has been interpreted as the product of information, motivation, and behavioral skills operating at the individual level [49]. Such an interpretation depicts the individual as the primary agent of behavior, and de-emphasizes social context.

As an analytic construct, social capital has been characterized as a property of individuals and of organizations. It has been used in the U.S. and Europe to examine the dynamics of civic engagement [50], the accomplishment of social action [51], and the production and reproduction of inequalities [52]. The ingredients of social capital (trust, cooperation, reciprocity, sociability) have been well-studied, and care has been taken to distinguish social capital from other forms of capital (e.g., economic, human, symbolic) [53]. Analysts agree on the characterization of social capital as a resource grounded in networks of social relationships. We define it as “resources accruing from a network of relationships that help individuals to solve problems and get things done.”

The concept of social capital adds considerable explanatory power to the study of HIV/AIDS in sub-Saharan Africa. It explains not only adherence success, but also the threat of stigma. Stigma is feared because it leads to social isolation, undermining relationships that are essential to survival. Avoiding HIV-related stigma can be understood as an effort to conserve social capital, a necessary resource in settings of poverty.

Relationships confer responsibility in addition to providing resources. Recipients of help must recognize what they receive and reciprocate. To ignore these responsibilities is to risk resentment on the part of helpers. Adherence allows patients to meet social responsibilities by preventing health decline and reducing the need for support. This creates a positive feedback loop in which social relationships help patients overcome economic barriers to sustained adherence. Adherence in turn fulfills responsibilities to others. Recognizing and fulfilling responsibilities to helpers through adherence strengthens social relationships and ensures more help will be available in the future.

Preservation of social capital lies at the heart of our explanation, but we recognize influences in other domains. These include: (1) social structure, e.g., patterns of inequality; (2) infrastructure, e.g. weaknesses in health systems; (3) culture, e.g., values, religious beliefs; and (4) individual experience and behavior, e.g., side effects, lack of information, psychological distress. We offer here a social relational theory to explain adherence differences between resource-rich and resource-poor settings, while acknowledging other kinds of determinants that merit further study.

Qualitative analyses contribute to quantitative research in medicine and public health by delineating useful constructs and testable hypotheses. In detailing the explanatory power of social capital, we point to its likely predictive value in examining hypotheses such as: Greater social capital will be associated with better ART access and adherence*.* Verification of such hypotheses will guide interventions to sustain adherence and improve treatment effectiveness.

A question that immediately arises is whether the benefits of social capital are sustainable? How long can patients realistically expect to receive resources from others who are struggling with resource scarcity themselves? Social capital, unlike many other forms of capital, increases with use [54]. However, while it may explain how economic obstacles to adherence have been overcome to date, it leaves the fundamental problem of poverty unaddressed. Caregiver efforts to compensate for the effects of poverty are not a substitute for affordable transportation, plentiful nutritious food, clean water, adequate living situations, and accessible and effective medical care. Eliminating underlying economic obstacles will reduce the strain on relationships and thus help to sustain both social capital and adherence.

We are not the first to address the social dimensions of adherence to ART in the context of international scale-up. A call for a biosocial framing laid out general principles of a socially grounded analysis and proposed relevant analytic concepts—social capital among them [55]. The importance of explaining adherence success in sub-Saharan Africa was stated specifically in a more recent account, which offered an anthropological history of treatment access and introduced “therapeutic citizenship” as an explanatory construct [56]. We propose an alternative construct emphasizing interpersonal relations, and add supporting data.

There are several limitations to our findings. First, African societies are highly heterogeneous. We collected data in urban and rural settings in East and West Africa. However, the extent to which these concepts explain adherence behavior in other African contexts will be an important area of future study. Second, we have developed a social explanation but left other domains of influence unexplored. As we develop theoretical models of adherence for resource-scarce locations, these types of influences should be more comprehensively represented. Third, while ART scale-up has been highly successful, the dynamics of adherence will likely evolve as patients confront long-term treatment. Finally, we draw upon social capital as an explanatory construct, but stop short of elaborating underlying cross-cultural differences in definitions of personhood [57].

### Conclusion

Like persons living with HIV/AIDS all over the world, sub-Saharan Africans adhere to ART because they want to be healthy. But the desire for health alone does not adequately explain adherence success. A more complete explanation highlights the role of social capital in relationships as a resource for prioritizing adherence and overcoming economic obstacles to care. Adherence preserves social capital by protecting relationships required for survival in settings of poverty. This may be what patients are referring to when they tell us they have “no choice” but to adhere.

## Abbreviations

ART: antiretroviral therapy
